# Vision-Related Quality of Life among Patients Attending the Diabetes and Eye Clinics in Kenyatta National Hospital, Kenya

**DOI:** 10.1155/2023/7809692

**Published:** 2023-01-17

**Authors:** Nerice Emade, Joseph Nyamori, Margaret Njuguna, Lucy Njambi, Stephen Gichuhi

**Affiliations:** ^1^MMed Ophthal, University of Nairobi, Nairobi, Kenya; ^2^Department of Ophthalmology, University of Nairobi, Nairobi, Kenya; ^3^Head Department of Ophthalmology, University of Nairobi, Nairobi, Kenya

## Abstract

**Objective:**

Our main objective was to determine the overall vision-related quality of life (VRQoL) among patients with diabetes mellitus attending the diabetes and eye clinics in Kenyatta National Hospital, Kenya.

**Design:**

Analytical cross-sectional study conducted in December 2020 setting: This study was performed at the Diabetes and Eye Clinics in Kenyatta National Hospital, the main national referral centre in Nairobi, Kenya. *Participants*. Using a purposive consecutive sampling method, we enrolled 100 participants, 50 with diabetic retinopathy and 50 without diabetic retinopathy. *Main Outcomes and Measures*. We compared the VRQoL of participants with diabetic retinopathy with those without diabetic retinopathy and assessed whether VRQoL worsened with increasing the severity of diabetic retinopathy. VRQoL was assessed using the World Health Organization/Prevention of Blindness and Deafness Vision Function-20 Questionnaire (VF-20). With this tool, the higher the mean score, the worse the quality of life. Diabetic retinopathy was graded using the Early Treatment of Diabetic Retinopathy Study. VRQoL trend with DR were analysed using the worse eye.

**Results:**

Participants with diabetic retinopathy had worse overall total VRQoL mean score (33.4, SD11.5) than those without (26.9, SD 4.7) in all domains; overall self-rating, 2.6 vs. 2.2, *p* < 0.001; general functioning, 18.0 vs. 14.7, *p*=0.005; psychosocial, 6.7 vs. 5.3, *p* < 0.001; and visual symptoms, 6.1 vs. 4.8, *p* < 0.001. VRQoL was worse with increasing severity of diabetic retinopathy in all domains moving from mild NPDR to moderate NPDR, severe NPDR and PDR, overall self-rating (2.2, 2.5, 3.5, 3.3; *p* < 0.001); visual symptoms (5.6, 5.6, 7.5, 7.4; *p*=0.002); psychosocial (5.7, 6.5, 6.0 8.8; *p*=0.004); and general functioning (15.7, 16.9, 17.5 23.6; *p*=0.014). Presence of DR, distance vision impairment, and diabetic macula oedema were associated with low overall self-rating. *Conclusion and Relevance.* Our findings underscore the need for interventions for early detection and management of diabetic retinopathy to prevent developing more advanced DR and its associated deterioration of VRQoL.

## 1. Introduction

Diabetes mellitus (DM) is a significant cause of morbidity and mortality as well as increased health-system cost [1]. Between 1990 and 2010, the global prevalence of DM tripled, and the incidence doubled [2]. The proportion of people living with diabetes is projected to increase by about one-third between 2025 and 2030 in high-income countries [3, 4]. The 2015 Kenyan STEP Survey showed that the nation-wide prevalence of DM was 2.4%, which was close to the 2.2% reported by the International Diabetes Federation [5, 6]. Diabetes mellitus can lead to several ocular complications such as diabetic retinopathy(DR), diabetic papillopathy, glaucoma, cataract, and ocular surface diseases [7]. DR is the most common microvascular complication affecting all the small retinal vessels by causing an increase in vascular permeability, ocular haemorrhages, lipid exudate, and the development of new vessels on the retina [8]. Therefore, much attention has been given to expanding the role of the current treatments (intravitreal pharmacotherapies, photocoagulation, and pars plans vitrectomy) for DR along with introducing novel therapies [9]. Novel therapies, including intravitreal human mesenchymal stem cell and intraoperative cold irrigating eye balanced salt solution, are currently being evaluated for the management of diabetic retinopathy and diabetic macula oedema [10–12].

Globally, about one-third of patients with DM aged 50 years and above have DR while about 10% have vision-threatening DR [13, 14]. A population-based study conducted between 2007 and 2008 in Kenya among adults aged ≥50 years reported that the prevalence of DR was 36% [15]. A follow-up study of the same cohort in 2013 found that the cumulative incidence of DM among previously nondiabetic participants aged ≥50 years was 61 per 1000 while that of DR was 15.8 per 1000 among those without DM before and 224.7 per 1000 among those with known DM before [16]. The Global Burden of Disease Study estimated that, between 1990 and 2020, the number of people aged ≥50 years who were blind from DR increased by 50% while the age-standardized prevalence of blindness due to DR increased by 15%. In Sub-Saharan Africa, the corresponding figures were 17% and 26%, respectively [17].

Recently, researchers have noted the significant role of quality of life (QoL) in the management of diabetes. Most studies investigating DR and QoL are from high-income countries and the questionnaires designed accordingly. Thus, they are not usually appropriate for all populations.

Different studies from around the world published inconsistent results on the QoL among patients with diabetes. A study assessing the impact of diabetic complications on health-related QoL using the SF-36 QoL questionnaire noted that DR had no effect on patients' QoL [18]. However, in a study aimed at assessing the effect of DR on QoL using the 26-domain retinopathy dependent QoL questionnaire, it was reported that vision loss due to DR had a significant impact on patients' QoL [19]. A study conducted in India assessing health-related and vision-related quality of life among patients with DR using the National Eye Institute 25-Item Visual Function Questionnaire concluded that QoL was worse in DR than in non-DR patients [20].

Several studies have reported a reduction in the vision-related quality of life in persons with DM and DR. However, little is known about the QoL of diabetics and specifically DR patients in Sub-Saharan Africa as well as in Kenya. To fill this gap, our study sought to use the WHO-PBD/VF-20 questionnaire which has been reported to be superior to most VRQoL questionnaires because it considers the mental and social impact as well as the vision-related activities [21]. VF-20 addresses 20 aspects of visual function grouped into 11 dimensions and 4 subscales. The WHO-PBD/VF-20 questionnaire has been validated for use in Kenya [22].

Understanding the VRQoL maybe important in assisting clinicians to manage DM and DR patients from a holistic point of view as well as guide policy makers and other stakeholders including patient support groups on national comprehensive diabetes care. In this regard, our aim was to investigate the VRQoL among people living with diabetes attending the Diabetes and Eye Clinics at the Kenyatta National Hospital using the WHO-PBD/VF-20 questionnaire.

## 2. Materials and Methods

### 2.1. Study Setting and Design

Kenyatta National Hospital (KNH) is in Nairobi, and it is the main national referral centre. This study was performed at the Diabetes and Endocrinology Centre of Excellence (diabetes clinic) as well as at the Eye Clinic in KNH. The diabetes clinic has a catchment population of over 3000 and the eye clinic over 300. This was a hospital-based analytic cross-sectional study conducted from 1^st^ December to 31^st^ December 2020.

### 2.2. Sampling Strategy and Recruitment

The minimum sample size for this study was derived from a previous study conducted in Nakuru using WHO/PBD-VF-20 questionnaire validated for use in Kenya [22]. Eligible participants were defined as those with either type 1 or type 2 DM for at least 5 years from the time of diagnosis. Participants included were aged 18 years and above, with or without diabetic macular oedema. A purposive consecutive sampling method was used, and we recruited 102 participants. Excluded from this study were diabetic patients with mental illness, gestational diabetes, as well as those with other visually impairing ocular morbidities such as glaucoma, retinal vascular occlusions, and optic neuritis.

In the diabetes clinic, patients with DM and fundus photographs indicating no DR were enrolled; meanwhile, those with DR were referred to the eye clinic for confirmation of retinopathy by a vitreoretinal specialist before enrollment.

### 2.3. Data Collection

Participants' blood pressure was measured upon arrival and their medical data on diabetes mellitus recorded from their files (duration of diabetes mellitus and latest glycated haemoglobin). The history of the course of microvascular complications such as the presence of hypertension was assessed. Best presenting visual acuity was determined for all participants using the E-charts for distant and near vision in a well-illuminated room. The distant E-charts were placed at 6 metres and the near E-charts at 40 cm.

In the eye clinic, slit-lamp examination was performed for all eligible participants to assess for the presence of cataract. Pharmacologic dilation of the participant's pupil was done using one drop of tropicamide 1%. Slit-lamp biomicroscopy, with a 90 dioptres lens, was used to diagnose diabetic retinopathy and diabetic macular oedema, which was then confirmed by the vitreoretinal specialist. The presence of dry eye syndrome was also assessed for all participants in both clinics using Schirmer's test strips and artificial tears only (Schirmer's test 1).

In the eye clinic, following confirmation of the presence of diabetic retinopathy, grading for each eye was done using the Early Treatment Diabetic Retinopathy Study (ETDRS) grading system. We assumed that patients' VRQoL will be driven by the worse eye with respect to diabetic retinopathy status. Diabetic macular oedema was defined as the presence of retinal thickening or hard exudates in the posterior pole within 500 mm of the fovea. Patients were with diabetes with any retinal thickening within 1/3 disc diameter (DD) of the centre of the macula, hard exudates within 1/3DD of the centre of macula with adjacent thickening, and retinal thickening ≥1DD of the centre of the macula.

In the diabetes clinic, all eligible participants had two fundus photographs taken per eye by a trained and validated technician using a nonmydriatic digital retinal camera (model CR-2AF). These images were digitally stored in a software. The assessment of cataract in this clinic was performed using the same camera. Participants were interviewed face-to-face by the principal investigator. Information was collected on demographic data, education, and employment status. For those who did not understand English, a translator was assigned to them. The WHO/PBD-VF-20 questionnaire was administered over a period of 15 minutes. We created a pictorial card illustrating scales 1 to 5 to help participants answer the WHO-PBD-VF-20 questionnaire.

### 2.4. Data Entry and Analysis

Data entry was done using Microsoft Excel and any inconsistency was corrected. Descriptive statistics was displayed using tables and figures. The descriptive data included the sociodemographic data, medical and ocular data, and VRQoL mean scores. Where data approximated a normal distribution, means and standard deviations were reported. Frequencies were reported with percentages and *p* values. The results of the student *t*-test comparing the mean score VRQoL between those with DR to those without DR were displayed in the same table, and *p* value <0.05 was considered statistically significant. We stratified the participants by degree of DR (using the ETDRS system) and tested for a linear trend of worsening VRQoL scores from baseline to the more advanced degrees of DR using the one-way ANOVA and post hoc tests. We conducted the analysis using the patient's worse eye in order to assess the association between ETDRS grades and QoL scores. We performed a multivariate analysis for all patients' overall eyesight rating adjusting for possible confounders such as age, sex, diabetic macula oedema, best presenting visual acuity, presence of cataract, dry eye syndrome, and duration of DM. The criteria used for explaining for good overall eyesight (mean score <3) and low overall eyesight (mean score ≥ 3) were based on the cut-off points for question 1 from the VFQ-20 questionnaire. Using the forward selection, a threshold of *p*=0.10 from univariate analysis was considered.

### 2.5. Ethics Statement

Ethical approval was obtained from the University of Nairobi Ethics and Research Committee, Kenyatta National Hospital (reference number: P356/07/2020), and the Kenyatta National Hospital administration. We obtained a written informed consent from all the participants. Participants and researchers' safety was ensured by adhering to the COVID-19 measures. Participants with DR were referred to a vitreoretinal (VR) specialist for confirmation of diagnosis and treatment.

## 3. Results

**Figure 1:**
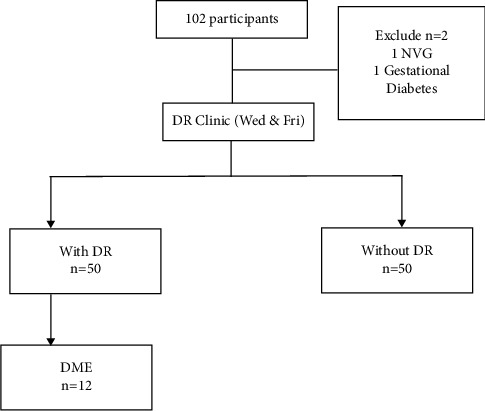
Result flowchart of participants attending the medical and eye clinics. DR: diabetic retinopathy; DME: diabetic macular enema; NVG: neovascular glaucoma.

We studied 100 participants, 50 with DR and 50 without DR. Among those with DR, only 12 participants were diagnosed with diabetic macule oedema (DME) (Figure 1). The mean age of participants with DR was 58.7 years and those without DR was 61.1 years (*p*=0.369). Most participants with DM (61%) and DR (66%) were females (Table 1). HbA1c was documented in 59% of participants (Table 2). Among patients with DR, majority were diagnosed with mild NPDR and a minimum number with severe NPDR. Patients with sight-threatening diabetic retinopathy received treatment in the form of anti-VEGF, laser photocoagulation, and/or vitrectomy (see supplementary table).

The composite VRQoL mean score for participants with DR was 33.4 (SD 11.5) while that of participants without DR was 26.9 (SD 4.7). For all the domains, participants with DR had significantly worse (higher) mean scores than those without DR (Table 3).

**Figure 2:**
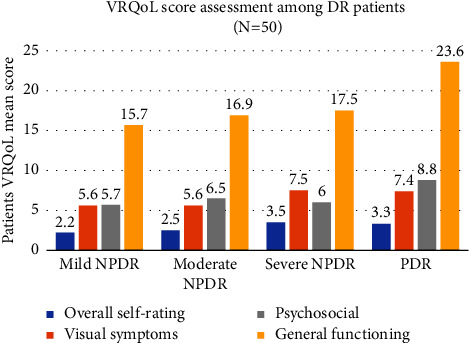
Graph illustrating VRQoL scores by ETDRS grades using patients' worse eye, Eye CLINIC, Kenyatta National Hospital. DR: diabetic retinopathy; NPDR: nonproliferative diabetic retinopathy; PDR: proliferative diabetic retinopathy; VRQoL: vision-related quality of life.

The trends in VRQoL mean score among participants with DR with different ETDRS grades using the better and worse eyes were similar. Using the worse eye, the VRQoL mean scores for the 4 domains increased with advanced DR with PDR having the highest mean scores and mild NPDR the lowest. The overall self-rating eyesight mean score (SD) for participants with PDR was slightly higher than those with mild NPDR (3.3 [0.8]; 2.2[0.5]). The VRQoL mean score (SD) for general functioning was highest among participants with PDR when compared to those with mild NPDR (23.6 [12.4] vs. 15.7 [3.8]) (Figure 2).

Marital status, BPVA, presence of cataract, and dry eye syndrome had statistically significant association with the diabetic retinopathy status (Tables 1 and 2). On multivariate analysis, the presence of DR, distance vision impairment, and the presence of DME were statistically associated with low overall QoL among all participants (Table 4).

## 4. Discussion

In our study, we found that the overall vision-related quality of life (VRQoL) among patients with DR was significantly poorer than those without DR using VF-20 questionnaire. This result was evident in all the four subscales of the VF-20 questionnaire with general functioning being the most affected followed by psychosocial functioning and visual symptoms. Patients' general vision perception was the least affected. Although our patients with DR perceived their overall eyesight as good, they still had difficulties in performing some daily activities. As regards patients' psychosocial status, some were worried about losing their sight, being a burden to others and being hesitant to participate in social gatherings. Similar results were documented in a previous study in India [20]. In contrast to a study by Wolf et al, the DM patients even without DR did not have any significant difficulty in seeing different colours [23]. As regards visual symptoms, we explored perceived ocular discomfort and difficulty seeing because of glare from bright light. This ocular discomfort had a significant impact on daily life of patients with DR when compared to those without DR. Contrary to Polack et al., our participants were able to report a significant effect of glare on their vision [22]. This discrepancy could be due to variations in study population and settings.

We also found that VRQoL in the patient's worse eye became poorer with increasing severity of the ETDRS grading. Using the better or the worse eye, patients with PDR had a significantly poorer perception of their general functioning followed by their psychosocial and visual symptoms. A study on the effect of DR and its severity on health-related quality of life in a population-based sample of Latinos with type 2 DM using the NEI VFQ-25 obtained similar results [24]. With the better eye, we noted that although the VRQoL again worsened with increasing severity, patients with no apparent DR had a poorer VRQoL when compared to those with mild NPDR. This was an unexpected, rare finding. In the multivariable analyses performed using general vision subscale as dependent variable in a binomial logistic regression model, higher mean scores were significantly associated with the presence of DR, distance vision impairment, and the presence of DME. Our results were comparable to those reported in a study conducted in India and in the USA, respectively, using the NEI VFQ-25 questionnaire [20, 23, 25].

Potential limitations of this study included patients' undetermined psychological status including the fear of being infected with COVID-19 while answering the questions might have affected the accuracy of answers, but the researcher had no means to control it. It is also important to note that we could not assess the effect of HbA1c on patients' perception of their general vision because of missing data in about a half of the patients.

## 5. Conclusion

In summary, quality of life was significantly lower in diabetics with DR when compared with those without DR with maximum effect seen on general functioning, psychosocial, visual symptoms, and general vision. Quality of life decreased with increasing severity of DR in the presence of DME and with distance vision impairment.

The results of this study are relevant because they demonstrate for the first time in our setting the negative influence of DR on patients' overall quality of life. Health professionals should be aware that quality of life is one of the primary objectives of diabetes treatment. Apart from the benefits in terms of visual outcomes, early identification and treatment of patients with DR would have a positive impact on the different dimensions of the patient's quality of life. However, the potential impact of the early diagnosis and treatment of DR on quality of life deserves the performance of specific intervention studies to address this issue. We also believe that there is a need for additional studies to conduct a linguistic and psychometric validation of new measures of quality of life and treatment satisfaction and to develop measurement tools that would allow the assessment of the impact of DR treatments on the patients in Kenya.

## Figures and Tables

**Table 1:** Participants' sociodemographic characteristics in relation to diabetes status at diabetes and eye clinics (*N* = 100).

Characteristics	*N* (%)	People with DR (*N* = 50)	People without DR (*N* = 50)	Crude OR (95% CI)	*p* value
		Mean (95% CI), *n* (%)	Mean (95% CI), *n* (%)		
Age (in years)					
≤30	1 (1.0)	1 (2.0)	0 (0.0)	—	0.605
31–40	6 (6.0)	2 (4.0)	4 (8.0)	0.7 (0.1–4.7)	
41–50	14 (14.0)	9 (18.0)	5 (10.0)	2.5 (0.6–10.3)	
51–60	27 (27.0)	12 (24.0)	15 (30.0)	1.1 (0.3–3.6)	
61–70	33 (33.0)	18 (36.0)	15 (30.0)	1.7 (0.5–5.2)	
>70	19 (19.0)	8 (16.0)	11 (22.0)	Reference	
Sex					
Male	39 (39.0)	17 (34.0)	22 (44.0)	0.7 (0.3–1.5)	0.306
Female	61 (61.0)	33 (66.0)	28 (56.0)	Reference	
Marital status					
Single	10 (10.0)	9 (18.0)	1 (2.0)	14.1 (1.5–137.3)	0.023
Married	72 (72.0)	34 (68.0)	38 (76.0)	1.4 (0.5–1.0)	
Divorced	18 (18.0)	7 (14.0)	11 (22.0)	Reference	
Education					
No formal schooling	9 (9.0)	4 (8.0)	5 (10.0)	0.6 (0.1–4.3)	0.927
Primary school	21 (21.0)	12 (24.0)	9 (18.0)	1.2 (0.4–3.9)	
Secondary	41 (41.0)	19 (38.0)	22 (44.0)	0.8 (0.3–2.1)	
Tertiary school	29 (29.0)	15 (30.0)	14 (28.0)	(Reference)	
Employment					
Salaried	24 (24.0)	9 (18.0)	15 (30.0)	0.6 (0.03–10.8)	0.122
Self-employed	46 (46.0)	21 (42.0)	25 (50.0)	0.8 (0.05–14.2)	
Retired	14 (14.0)	11 (22.0)	3 (6.0)	3.7 (0.2–77.6)	
Unemployed	16 (16.0)	9 (18.0)	24 (48.0)	(Reference)	

**Table 2:** Participants' ocular and medical characteristics in relation to diabetes status at diabetes and eye clinics (*N* = 100).

Characteristics	*N* (%)	People with DR (*N* = 50)	People without DR (*N* = 50)	*p* value
		*n* (%), mean (SD)	*n* (%), mean (SD)	
BPVA				
Normal (≥6/12)	63 (63.0)	31 (62.0)	32 (84.0)	0.031
Mild (<6/12 to 6/18)	13 (13.0)	10 (20.0)	3 (6.0)	
Moderate (<6/18 to 6/60)	10 (10.0)	5 (10.0)	5 (10.0)	
Severe (<6/60 to 3/60)	1 (1.0)	1 (2.0)	0 (0.0)	
Blindness (<3/60)	3 (3.0)	3 (6.0)	0 (0.0)	
Presence of cataract (*n* = 43)				
Unilateral	6 (14.0)	03 (50.0)	03 (50.0)	0.008
Bilateral	37 (86.0)	25 (67.6)	12 (32.4)	0.024
Presence of DES				
Yes	81 (81.0)	47 (94.0)	34 (68.0)	0.002
No	19 (19.0)	3 (6.0)	16 (32.0)	
Spectacles use				
None	73 (73.0)	38 (76.0)	35 (70.0)	0.648
Distant correction only	8 (8.0)	2 (4.0)	6 (12.0)	
Near correction only	8 (8.0)	2 (4.0)	6 (12.0)	
Near correction only	9 (9.0)	4 (8.0)	5 (10.0)	
Distant and near correction	10 (10.0)	6 (12.0)	4 (8.0)	
Duration of DM (years), mean (SD)	100	113.6 ± 6.9	13.7 ± 6.2	0.928
Latest HbA1c (g/dl), mean (SD)	59	9.1 ± 3.5	7.5 ± 3.0	0.072

BPVA: best presenting visual acuity, VA: visual acuity, DM: diabetes mellitus, HbA1c: glycated haemoglobin, and LogMAR: logarithm of minimum angle of resolution.

**Table 3:** Vision-related quality of life mean scores among patients attending the diabetes and eye clinics.

Subscales of WHO-PBD_VF-20 questionnaire	DR (*n* = 50), mean (SD)	No DR (*n* = 50), mean (SD)	*p* value
Overall self-rating	2.6 (0.7)	2.2 (0.4)	<0.001
General functioning	18.0 (7.6)	14.7 (3.3)	0.005
Distance vision difficulty	7.7 (3.5)	6.0 (1.4)	
Near vision difficulty	7.7 (3.1)	6.4 (1.7)	
Role limitations	1.5 (0.8)	1.2 (0.5)	
Colour vision difficulty	1.1 (0.6)	1.0 (0.0)	
Psychosocial	6.7 (2.6)	5.3 (1.0)	<0.001
Mental well-being	3.9 (1.4)	3.2 (1.0)	
Social functioning limitations	1.5 (0.8)	1.1 (0.2)	
Dependency	1.3 (0.7)	1.0 (0.1)	
Visual symptoms	6.1 (1.5)	4.8 (1.2)	<0.001
Light/dark adaptation	2.2 (0.8)	1.6 (0.6)	
Ocular pain/discomfort	2.0 (0.5)	1.6 (0.5)	
Glare	1.9 (0.8)	1.6 (0.6)	

WHO-PBD VF-20 questionnaire: World Health Organization Prevention of Blindness and Deafness Visual Function-20 Questionnaire.

**Table 4:** Binominal logistic regression of general vision for all DM patients (*N* = 100).

Variables	Low overall QoL (*n*, %)	Good overall QoL (*n*, %)	Crude Odds ratio (95% CI)	*p* value	Adjusted Odds ratio (95% CI)	*p* value
DR						
Yes	26 (78.8)	24 (35.8)	6.6 (2.29–19.37)	0.0001	5.8 (2.1–15.9)	0.001
No	7 (21.2)	43 (64.2)	Reference		Reference	
Age in years *(mean, sd)*	60.1 (2.19)	59.8 (1.70)	—	0.938	—	—
Sex						
Female	22 (66.7)	39 (58.2)	1.4 (0.6–3.5)	0.457	—	—
Male	11 (33.3)	28 (41.8)	Reference			
Distance vision impairment						
Yes (VA worse than 6/12)	22 (66.7)	19 (28.4)	5.1 (1.9–13.3)	0.0003	4.3 (1.6–11.2)	0.003
No (VA better or equal to 6/12)	11 (33.3)	48 (71.6)	Reference		Reference	
Diabetic macula oedema						
Yes	10 (30.3)	2 (4.5)	9.3 (2.1–40.8)	0.028	4.8 (1.1–21.3)	0.037
No	23 (69.7)	67 (95.5)	Reference		Reference	
Cataract in any eye						
Yes	19 (57.6)	22 (32.8)	2.8 (1.1–6.7)	0.018	2.6 (0.9–8.1)	0.109
No	14 (42.4)	45 (67.2)	Reference		Reference	
Dry eye syndrome						
Yes	30 (90.9)	51 (76.1)	3.1 (0.8–11.7)	0.078	—	—
No	3 (9.1)	16 (23.9)	Reference			
Systemic comorbidity						
Yes	24 (72.7)	55 (82.1)	0.6 (0.2–1.6)	0.282	—	—
No	9 (27.3)	12 (17.9)	Reference			

## Data Availability

The data used to support the findings of this study are available from the corresponding author upon request.

## Supplementary Materials

Supplementary Materials The supplementary table describes the diabetic retinopathy status among patients attending the eye clinics using their better and worse eyes. As illustrated in the table, using the patient's better or worse eyes, majority were found to have mild NPDR (44%) and a minimum number had severe NPDR (4%). Again, patients with sight-threatening diabetic retinopathy received treatment in the form of anti-VEGF (36.4%), laser photocoagulation + vitrectomy (18.1%), or anti-VEGF + laser photocoagulation (9.1%).

## References

[1] Global Burden of Metabolic Risk Factors for Chronic Diseases Collaboration (2014). Cardiovascular disease, chronic kidney disease, and diabetes mortality burden of cardiometabolic risk factors from 1980 to 2010: a comparative risk assessment. *Lancet Diabetes & Endocrinology*.

[2] Gregg E. W., Li Y., Wang J. (2014). Changes in diabetes-related complications in the United States, 1990-2010. *New England Journal of Medicine*.

[3] Murphy R. P. (1995). Management of diabetic retinopathy. *American Family Physician*.

[4] Ministry of Public Health (2017). *Guidelines for Screening and Management of Diabetic Retinopathy in Kenya*.

[5] Mohamed S. F., Mwangi M., Mutua M. K. (2018). Prevalence and factors associated with pre-diabetes and diabetes mellitus in Kenya: results from a national survey. *BMC Public Health*.

[6] Idf (2015). IDF Diabetes Atlas. https://www.diabetesatlas.org/upload/resources/previous/files/7/IDF%20Diabetes%20Atlas%207th.pdf.

[7] Threatt J., Williamson J. F., Huynh K., Davis R. M., Hermayer K. (2013). Ocular disease, knowledge and technology applications in patients with diabetes. *The American Journal of the Medical Sciences*.

[8] Singh P. P., Mahadi F., Roy A., Sharma P. (2009). Reactive oxygen species, reactive nitrogen species and antioxidants in etiopathogenesis of diabetes mellitus type-2. *Indian Journal of Clinical Biochemistry*.

[9] Vaziri K., Schwartz S. G., Relhan N., Kishor K. S., Flynn H. W. (2015). New therapeutic approaches in diabetic retinopathy. *The Review of Diabetic Studies : Regional Development Studies*.

[10] Scalinci S. Z., Scalinci L., Scorolli G. (2011). Potential role of intravitreal human placental stem cell implants in inhibiting progression of diabetic retinopathy in type 2 diabetes: neuroprotective growth factors in the vitreous. *Clinical Ophthalmology*.

[11] Meduri A., Oliverio G. W., Bergandi L. (2021). Role of cold balanced salt solution (BSS) in the prophylaxis of cystoid macular edema after cataract surgery: a prospective randomized study. *Clinical Ophthalmology*.

[12] Meduri A., Oliverio G. W., Trombetta L., Giordano M., Inferrera L., Trombetta C. J. (2021). Optical coherence tomography predictors of favorable functional response in naïve diabetic macular edema eyes treated with dexamethasone implants as a first-line agent. *Journal of Ophthalmology*.

[13] Teo Z. L., Tham Y.-C., Yu M. (2021). Global prevalence of diabetic retinopathy and projection of burden through 2045. *Ophthalmology*.

[14] Yau J. W. Y., Rogers S. L., Kawasaki R. (2012). Global prevalence and major risk factors of diabetic retinopathy. *Diabetes Care*.

[15] Mathenge W., Bastawrous A., Peto T. (2014). Prevalence and correlates of diabetic retinopathy in a population-based survey of older people in Nakuru, Kenya. *Ophthalmic Epidemiology*.

[16] Bastawrous A., Mathenge W., Wing K. (2017). The incidence of diabetes mellitus and diabetic retinopathy in a population-based cohort study of people age 50 years and over in Nakuru, Kenya. *BMC Endocrine Disorders*.

[17] Gbd 2019 (2021). GBD 2019 blindness and vision impairment collaborators, vision loss expert group of the global burden of disease study. Causes of blindness and vision impairment in 2020 and trends over 30 years, and prevalence of avoidable blindness in relation to VISION 2020: the right to sight: an analysis for the global burden of disease study. *Lancet Global Health*.

[18] Lloyd A., Sawyer W., Hopkinson P. (2001). Impact of long-term complications on quality of life in patients with type 2 diabetes not using insulin. *Value in Health*.

[19] Woodcock A., Bradley C., Plowright R., ffytche T., Kennedy-Martin A., Hirsch A. (2004). The influence of diabetic retinopathy on quality of life. *Patient Education and Counseling*.

[20] Pereira D. M., Shah A., D’Souza M. (2017). Quality of life in people with diabetic retinopathy: Indian study. *Journal of Clinical and Diagnostic Research: Journal of Clinical and Diagnostic Research*.

[21] WHO (2003). Consultation On Development of Standards for Characterization of Vision Loss and Visual Functioning: Geneva. https://apps.who.int/iris/handle/10665/68601.

[22] Polack S., Kuper H., Mathenge W., Fletcher A., Foster A. (2007). Cataract visual impairment and quality of life in a Kenyan population. *British Journal of Ophthalmology*.

[23] Wolff B. E., Bearse M. A., Schneck M. E. (2015). Color vision and neuroretinal function in diabetes. *Documenta Ophthalmologica*.

[24] Mazhar K., Varma R., Choudhury F., McKean-Cowdin R., Shtir C. J., Azen S. P. (2011). Severity of diabetic retinopathy and health-related quality of life. *Ophthalmology*.

[25] Hariprasad S. M., Mieler W. F., Grassi M., Green J. L., Jager R. D., Miller L. (2008). Vision-related quality of life in patients with diabetic macular oedema. *British Journal of Ophthalmology*.

