# Supplementary material for: Vision-Related Quality of Life among Patients Attending the Diabetes and Eye Clinics in Kenyatta National Hospital, Kenya
Source: J Ophthalmol. 2023 Jan 17;2023:7809692. doi: 10.1155/2023/7809692 (PMC9873415; doi:10.1155/2023/7809692)
Supplement: Supplementary Materials — The supplementary table describes the diabetic retinopathy status among patients attending the eye clinics using their better and worse eyes. As illustrated in the table, using the patient's better or worse eyes, majority were found to have mild NPDR (44%) and a minimum number had severe NPDR (4%). Again, patients with sight-threatening diabetic retinopathy received treatment in the form of anti-VEGF (36.4%), laser photocoagulation + vitrectomy (18.1%), or anti-VEGF + laser photocoagulation (9.1%). [file 7809692.f1.docx]

**Table (Suppl 1): Diabetic retinopathy status among patients attending the Eye clinics using their better and worse eye, Kenyatta National Hospital, Kenya.**

| Variables | Patient’s Better Eye | Patient’s Worse Eye |
| --- | --- | --- |
|  | N=50  n (%) | N=50  n (%) |
| ETDRS grade |  |  |
| No apparent DR | 2 (4.0) | - |
| Mild NPDR | 25 (50.0) | 22 (44.0) |
| Moderate NPDR | 12 (24.0) | 14 (28.0) |
| Severe NPDR | 2 (4.0) | 2 (4.0) |
| PDR | 9 (18.0) | 12 (24.0) |
| DME |  |  |
| Present | 12 (24.0) | 12 (24.0) |
| Not present | 38 (76.0) | 38 (76.0) |
| Ever been treated for DR/ Maculopathy |  |  |
| Yes | 22 (44.0) | 22 (44.0) |
| No | 28 (56.0) | 28 (56.0) |
| Type of treatment given |  |  |
| Anti-VEGF | 8 (36.4) | 8 (36.4) |
| Laser photocoagulation | 8 (36.4) | 8 (36.4) |
| Anti-VEGF + laser photocoagulation | 2 (9.1) | 2 (9.1) |
| Laser photocoagulation + Vitrectomy | 4 (18.1) | 4 (18.1) |

DR: Diabetic retinopathy, NPDR: non-proliferative diabetic retinopathy, PDR: proliferative diabetic retinopathy, VEGF: vascular endothelial growth factor, DME: diabetic macular oedema, ETDRS: early treatment diabetic retinopathy study
